# Comment on Herring et al. The Use of “Retardation” in FRAXA, FMRP, FMR1 and Other Designations. *Cells* 2022, *11*, 1044

**DOI:** 10.3390/cells11121937

**Published:** 2022-06-16

**Authors:** Elspeth Bruford, on behalf of the HUGO Gene Nomenclature Committee (HGNC)

**Affiliations:** 1 Department of Haematology, University of Cambridge School of Clinical Medicine, Cambridge CB2 0PT, UK; 2 HUGO Gene Nomenclature Committee, EMBL-EBI, European Molecular Biology Laboratory, Wellcome Genome Campus, Hinxton CB10 1SD, UK; hgnc@genenames.org

**Keywords:** gene nomenclature, HGNC, fragile X syndrome, FMR1, FRAXA

## Abstract

This commentary is written in response to the recent article from Herring et al., discussing the eradication of the offensive term “retardation” from gene nomenclature. We discuss the work of the HUGO (Human Genome Organisation) Gene Nomenclature Committee (HGNC) and outline the steps already taken to remove this term from our gene names. We also highlight the latest nomenclature changes made as a result of discussions with the authors and agreement with the European Fragile X Network.

## 1. Introduction

Using standardized gene symbols and names is critical for effective communication, and as genomics becomes more and more routinely implemented in the clinic, using a common language becomes ever more important. To this end we, the HUGO Gene Nomenclature Committee (HGNC, www.genenames.org), were pleased to engage with the authors Herring et al., following their recent publication in Cells [1] in which they argued that all references to ‘retardation’ should be removed from gene nomenclature; their proposal included removing the letter ‘R’ within gene symbols in which it once stood for ‘retardation’. As this led to a fruitful exchange, we hoped it would be useful for readers to learn about the outcomes of our discussions and also to find out more about the work of the HGNC, which has been the sole body responsible for naming human genes since 1979.

## 2. FRAXA

The HGNC assigns names and symbols not only for protein-coding genes, pseudogenes, and RNA genes but also for a limited number of other “locus types”, such as fragile sites and, previously, human phenotypes. The symbol “FRAXA” quoted in the article from Herring et al., is a symbol for a fragile site, a region of genomic instability (i.e., not an actual gene). As the fragile site symbol *FRAXA* has also confusingly been used in the literature to refer to the gene underlying “fragile X syndrome”, we also list “FRAXA” as an alias symbol for *FMR1*. Indeed, this fragile site on the X chromosome is the derivation of the name of “fragile X syndrome”, which was originally eponymously referred to as “Martin-Bell syndrome” after clinicians who described the condition in a large family in the 1940s [2]. In the case of *FRAXA*, which was first approved by the HGNC as a fragile site symbol in 1986 [3], this “fragility” is due to an unstable repeat of three nucleotides (CGG) which is found in the 5′ untranslated region of the *FMR1* gene. A considerable expansion of this repeat can silence the expression of *FMR1* [4], potentially resulting in the range of characteristics associated with fragile X syndrome.

We were only too happy to update the full name of the *FRAXA* fragile site when this was brought to our attention by Herring et al., and to acknowledge that we had unfortunately overlooked checking the names of fragile site loci when removing the term “retardation” from our nomenclature. Between 2015 and 2019, we removed uses of “retardation” from our approved gene names (e.g., *FMR1*, *ATRX*, and *AMMECR1*), in line with movements such as Rosa’s Law and “Spread the Word to End the Word” [5,6], as we appreciated how potentially offensive this term can be, especially in a clinical setting.

## 3. FMR1 and FMRP

As Herring et al., noted, we stated in our 2020 article discussing our current gene nomenclature guidelines [7] that we now strive to ensure that approved gene symbols and names “Must not be offensive or pejorative (ideally in any language)”. However, we also stated that “the stability of gene symbols, particularly those associated with disease, is now a key priority for the HGNC. Nevertheless, novel information can be encapsulated in the gene name without changing the gene symbol”.

With this in mind, we did not agree with the authors’ proposed change of gene symbol from *FMR1* to FX1 or FXTR1, especially when the *FMR1* symbol is unique and so deeply entrenched in the biomedical literature, having been used in over 4000 publications to date. When the *FMR1* symbol was originally approved, it had the accompanying name “fragile X mental retardation 1”, but in 2019, we updated this name to “FMRP translational regulator 1”, both to remove the pejorative term and to provide some functional information about the encoded protein. Unfortunately, the original gene name is still widely used in the literature; non-approved gene symbols and names are used in numerous peer-reviewed scientific articles because the HGNC has no control whatsoever over what authors choose to publish. All we can do is freely provide the nomenclature and hope that authors, researchers, clinicians, reviewers, editors, and journals will actually use it or enforce its use. Indeed, we sincerely hope that articles such as this will help our efforts to ensure that approved gene nomenclature is more widely implemented.

In our subsequent correspondence, Herring et al., questioned what the letters “MR” stood for in the “FMRP translational regulator 1” gene name and voiced concerns that a link to the term “mental retardation” was still implied. We understood their concerns and went back to the literature to look for further information on the functions of the *FMR1* gene product. This led us to a tranche of papers that discuss how the encoded protein acts as a messenger ribonucleoprotein, a cytoplasmic RNA binding protein that regulates translation [8]. Therefore, we proposed the new gene name “Fragile X messenger ribonucleoprotein 1”, where messenger can stand for the “M” and ribonucleoprotein for the “R” in the symbol, thus breaking any implied link to “mental retardation” and removing the need for a change to the established *FMR1* symbol. We were delighted when, after deliberation by members of the European Fragile X Network, they agreed that the new name, without a need for a change of gene symbol, is a good solution. This name change has been implemented not only for the human gene, but also for the equivalent orthologous genes in other vertebrate species, for example, in mice [9] and zebrafish [10], and following discussions with the UniProt resource, it will also be listed as their “recommended protein name”.

At this point we should clarify that “FMRP”, the acronym widely used for the protein encoded by *FMR1*, is not an “approved” protein symbol—there is no single authority for the naming of human proteins, and FMRP is simply an acronym that has proven popular in the literature. As such, we have no control over what FMRP is meant to stand for—and indeed, we always recommend using the gene symbol in italics to refer to the gene (*FMR1*) and using the non-italicised symbol to refer to the encoded protein (FMR1). However, we realise that usage of FMRP will undoubtedly continue, so we recommend its use with the accompanying name of “Fragile X messenger ribonucleoprotein” in line with the new *FMR1* gene name.

## 4. AFF Family

Herring et al., also voiced concerns about the “FMR2” symbol: again, this is not an approved gene symbol, but in this case, it is what we term a “previous symbol” because it was approved until 2005 when it was replaced with the *AFF2* gene symbol. There are four homologous genes in the “AFF” family, *AFF1*–*4*. The AFF root symbol is derived from AF4/AF-4, standing for “ALL1-fused gene from chromosome 4” (ALL-1 is an alias for the *KMT2A* gene—also known as MLL1—because “AF4”, now approved as *AFF1*, was recurringly found in chromosomal translocations with *KMT2A* in acute lymphoid leukaemia [11]), combined with the F from FMR2. The AFF family members all had the gene name “AF4/FMR2 family member #”, but we could appreciate that referencing “FMR2” in these gene names was not ideal. Again, the literature surrounding these genes provided an alternative name for this family that was already used in a number of publications: the “ALF transcription elongation factors” [12,13,14]. While the ALF acronym is derived from the gene aliases/previous symbols “AF4” (approved as *AFF1*), “LAF4” (approved as *AFF3*), and “FMR2” (approved as *AFF2*), we feel that this is suitably removed from the original derivation of FMR2 and hence has no negative associations.

## 5. Summary of Changes

To summarise the changes we have made:

- The name associated with the *FRAXA* fragile site symbol has been updated to “Fragile X Site, Folic Acid Type, Rare, Fra(X) (Q27.3) A”;
- The name associated with the *FMR1* gene symbol has been updated to “Fragile X messenger ribonucleoprotein 1”;
- We advise that the FMRP protein alias is referred to as “Fragile X messenger ribonucleoprotein”;
- We advise consistent use of the symbol *AFF2* for the gene previously approved as *FMR2*;
- All members of the AFF family (*AFF1*-*4*) now have the new root name “ALF transcription elongation factor #”.

As with the update to the *FMR1* gene name, these changes will be adopted for orthologous genes across vertebrates; however, it is worth bearing in mind that it can take time for changes to percolate throughout all online resources, as they all have different update cycles. In the meantime, we encourage the active use of the new nomenclature in all forthcoming publications and presentations discussing these genes.

## 6. Previous Gene Symbols and Names

In their publication, Herring et al., also referenced a number of previous “gene names” that had included pejorative terms but are now listed in the “previous names” field in entries for known genes, such as *AP1S2*, *RAB39B*, *ARX*, *HDAC4*, and *PQBP1*. These “previous names” were not intended to refer to genes but rather to phenotypes, which the HGNC used to name; this activity has since been passed on to the OMIM database [15]. Previously, if the causative gene for a phenotype with an HGNC symbol and name was discovered, the HGNC would then merge the phenotype entry (e.g., *WSN*, “Waisman syndrome”) into the entry for the causative gene (e.g., *RAB39B*), and hence, these phenotype symbols and names are listed as “previous symbols” and “previous names” in some gene entries.

It should be noted that our resource acts as an archive of gene nomenclature, and numerous publications in the public domain will, albeit regrettably, continue to use previous gene and phenotype names despite our best efforts; therefore, to break the link between the genes and these previous names would leave the way open to considerable confusion. Hence, we do not believe that the previous names should be erased any more than all the publications using the term “mental retardation” should be expunged from the literature—rather, we consign them to the archival fields and encourage the use of the current approved nomenclature.

## 7. Conclusions

We are confident that gene naming has progressed considerably in the five decades that the HGNC has been operating. As genes are becoming more and more commonly discussed outside the spheres of biomedical research, we are increasingly interested in engaging more with clinicians, nurses, genetic counsellors, and patient support groups to find appropriate solutions to naming issues, as we have done in this case. For example, in 2020, we were delighted to have the support of the Barth Syndrome Foundation [16] when we proposed updating the symbol for the gene encoding the protein tafazzin from the confusing “*TAZ*” symbol (also commonly used in the literature for the unrelated *WWTR1* gene) to simply “*TAFAZZIN*” [17]. While this may seem to contradict our wish for symbol stability, in this instance, it changed what had been a confusing symbol that was being used for more than one gene (*TAZ*) to a unique symbol that is instantly recognisable and that references the already widely used protein name (*TAFAZZIN*).

Ultimately, we aim for everyone interested in genes to speak the same language, and we welcome feedback and input wherever possible.
